# Photoinactivation of influenza viruses by modulated indoor daylight spectrum and intensity

**DOI:** 10.1128/aem.00973-25

**Published:** 2025-10-02

**Authors:** Michael Zhao, Kinga Vojnits, Man In Lam, Sam Yeo, Piers MacNaughton, Sepideh Pakpour

**Affiliations:** 1 Faculty of Applied Science, School of Engineering, University of British Columbia 685573, Kelowna, British Columbia, Canada; 2 SeeAir Inc., Cambridge, Massachusetts, USA; Centers for Disease Control and Prevention, Atlanta, Georgia, USA

**Keywords:** virus infectivity, virus genomic stability, electrochromic windows, indoor daylight, bacteriophage MS2, influenza B virus, influenza A virus, influenza

## Abstract

Influenza claims over 290,000 lives annually, and the annual flu season remains a major public health challenge. Within the domain of health-focused architectural design, indoor daylight emerges as a potential shield against microbial threats. Yet, a significant gap in our understanding remains: the effect of indoor daylight on viruses. To bridge this knowledge gap, we used a living lab setup to investigate the impact of modulated indoor daylight on the stability of genomic material and persistence of infectivity of enveloped influenza A and B viruses, along with the non-enveloped bacteriophage MS2 on an environmental surface of glass. Indoor daylight modulation showed capacity for viral inactivation with differential activity depending on spectrum, intensity, and duration of exposure. Intensity was the most impactful, significantly reducing the infectivity of influenza A virus and both the infectivity and amount of recoverable genomic material of influenza B virus following 8 h of exposure. Even at low-intensity light, comparable to light passing through traditional blinds, spectrum-modulated, blue-enriched light significantly reduced the infectivity of influenza A and B within 8 h. Moreover, for all viruses, the infectivity declined before the stability of genomic material, suggesting a mechanism of photoinactivation via the disruption of viral proteins involved in infection. This research emphasizes the paramount importance of considering daylighting conditions as a strategic approach to control infectious disease transmission in built environments. Our findings not only underscore this significance but also offer innovative pathways to transform indoor spaces into safer, healthier environments for everyone.

**IMPORTANCE:**

This study examined the interplay between indoor daylighting and viruses, specifically influenza A, influenza B, and MS2 bacteriophage in a simulated indoor environment on a surface material of glass. It demonstrated that indoor daylight modulation was able to inactivate influenza A and B following 8 h of exposure at high-intensity and low-intensity blue-enriched light; however, the stability of genomic material of influenza A was unaffected until at least 24 h of exposure. These results, which focus on differences between stability of genomic material and infectivity, provide deeper insight into viral photoinactivation mechanisms, and the use of a living-lab setup lays the foundation for a framework for healthy building design using indoor daylight modulation for infection control.

## INTRODUCTION

Influenza remains a significant global health and economic burden, with an estimated 1 billion infections annually, including 3–5 million cases of severe illness and 294,000–518,000 respiratory deaths worldwide (1, 2). Beyond the direct health toll, influenza infections impose substantial economic costs, contributing to an $11.2 billion annual burden in the United States alone through direct medical expenses and lost productivity (3). Seasonal epidemics during the winter months, known as the “flu season,” stretch from October to May and peak in February (4, 5). These outbreaks are driven by two primary types of influenza viruses: influenza A virus (IAV) and influenza B virus (IBV), which together account for the majority of global cases (4). While IAV has historically received greater attention due to its association with pandemics (4, 6), IBV also plays a critical role, contributing to 25% of all influenza virus (IV)–related mortality in the United States (7) and a similar proportion of the economic burden (6). Notably, IBV has recently gained attention for its disproportionate impact on children (6, 8). Despite advances in vaccines and antiviral medications, interventional strategies remain insufficient to fully control influenza’s impact. Vaccines have significantly reduced the disease burden; however, mismatches between vaccine strains and circulating viruses limit their efficacy, which averages 50–60% even in well-matched seasons (7, 9–11). Antiviral drugs, such as oseltamivir and zanamivir, provide critical treatment options but are primarily reserved for severely ill or immunocompromised individuals, and their high costs contribute to the economic burden of the disease (3). Furthermore, these strategies have not resolved the persistently high infection rates during flu season, underscoring the urgent need for innovative approaches to mitigate transmission and reduce influenza’s impact on human health and the economy.

The heightened transmission of influenza during flu season is closely linked to increased time spent indoors, where environmental factors play a critical role in facilitating the spread of airborne pathogens (12, 13). Poorly ventilated indoor spaces create conditions that amplify the transmission of airborne particles (14, 15), and interventions for indoor infection control have increasingly focused on addressing these factors to reduce transmission risks. Historically, IV transmission has been studied based on three routes: droplets (>10 µm) that deposit in the upper respiratory tract, droplet nuclei/aerosols (<5 µm) that reach the lungs, and contact transmission via direct or indirect contact with contaminated surfaces or fomites (16, 17). More recently, these pathways have been categorized into two major modes of transmission: (1) airborne transmission, encompassing both inhalation of aerosols and direct deposition of droplets, and (2) contact transmission, which includes direct physical contact and indirect transmission via contaminated surfaces (18). Despite these classifications, the relative contributions of each route to overall disease spread remain under debate (16, 19).

Current indoor infection control strategies target these transmission routes through measures such as mask-wearing and chemical disinfection (20, 21). However, these approaches face several limitations, including issues with social compliance (22), potential long-term health effects from chemical exposure, such as increased rates of respiratory disease (21, 23), and the need for frequent reapplication due to rapid surface recontamination (24). Ultraviolet (UV) light has long been studied as a tool for infection control, with UVC radiation demonstrating powerful germicidal properties (25). Ultraviolet germicidal irradiation (UVGI) has been shown to effectively photoinactivate a range of pathogens, including viruses, both on surfaces and in aerosols (26–29). However, the use of UVC indoors requires adherence to strict safety guidelines due to its potential to cause direct harm to human cells. Consequently, UVGI is primarily employed in air ducts or upper-room installations, where it disinfects air with minimal occupant exposure (30–32). Additionally, UVC’s known effects on material degradation and potential impacts on indoor air quality have raised concerns about its broader applicability in indoor environments (33). In response to these limitations, attention has shifted toward irradiation methods that use visible light, such as antimicrobial blue light (ABL) in the 400–500 nm range. ABL has recently shown promise for its bactericidal and fungicidal properties (34, 35). Emerging studies also suggest that ABL can photoinactivate enveloped viruses, including influenza, though non-enveloped viruses appear more resistant (36–41). Unlike UV radiation, ABL does not harm human cells or materials and is abundant in natural sunlight, allowing it to pass through traditional windows, a property that UVC lacks due to filtering. Despite these advantages, the potential of ABL to influence the persistence and infectivity of viruses in indoor environments remains poorly explored, presenting a critical gap in current research.

Windows, as the primary modulators of indoor daylight, play a significant role in shaping the spectrum and intensity of light within indoor spaces (42, 43). These variations in light composition can passively influence the indoor environment in ways that may affect viral genomic stability and activity. Traditional windows, however, often reduce the availability of antimicrobial blue light (ABL) due to common practices, such as using blinds to mitigate glare, regulate temperature, and ensure privacy (44). These practices, while addressing comfort, inadvertently limit exposure to light wavelengths that could have implications for pathogen control. Advanced technologies, such as electrochromic (EC) windows, offer the ability to dynamically adjust indoor light composition by modulating daylight intensity and spectral properties (45). This modulation has implications beyond energy efficiency (42, 43), with potential impacts on occupant health and environmental disinfection (46–48). EC windows can enrich the indoor spectrum with ABL, offering an opportunity to explore how daylight composition might influence the genomic stability and infectivity of pathogens. Previous studies have demonstrated that spectral modulation through EC windows can exert photoactive disinfectant effects on bacterial and fungal pathogens (35). However, their influence on viruses, including influenza viruses (IVs), remains underexplored, underscoring the need for focused investigation.

To address this knowledge gap, we designed experiments using an environmental chamber to simulate indoor conditions with controlled daylight profiles. Simulated sunlight was passed through EC windows at two settings: electrochromic clear (ECC), designed to mimic natural sunlight through traditional windows, and electrochromic tinted (ECT), mimicking the reduced light intensity of sunlight filtered through blinds but enriched with ABL. These profiles were compared to conditions with traditional blinds to assess their effects on the genomic stability and infectivity of enveloped viruses (influenza A and influenza B) and a non-enveloped virus (bacteriophage MS2) applied as droplets on glass surfaces. This study aimed to evaluate whether modulating indoor light through EC windows could passively influence viral characteristics within indoor environments.

## MATERIALS AND METHODS

### Experimental chamber setup

**Fig 1:**
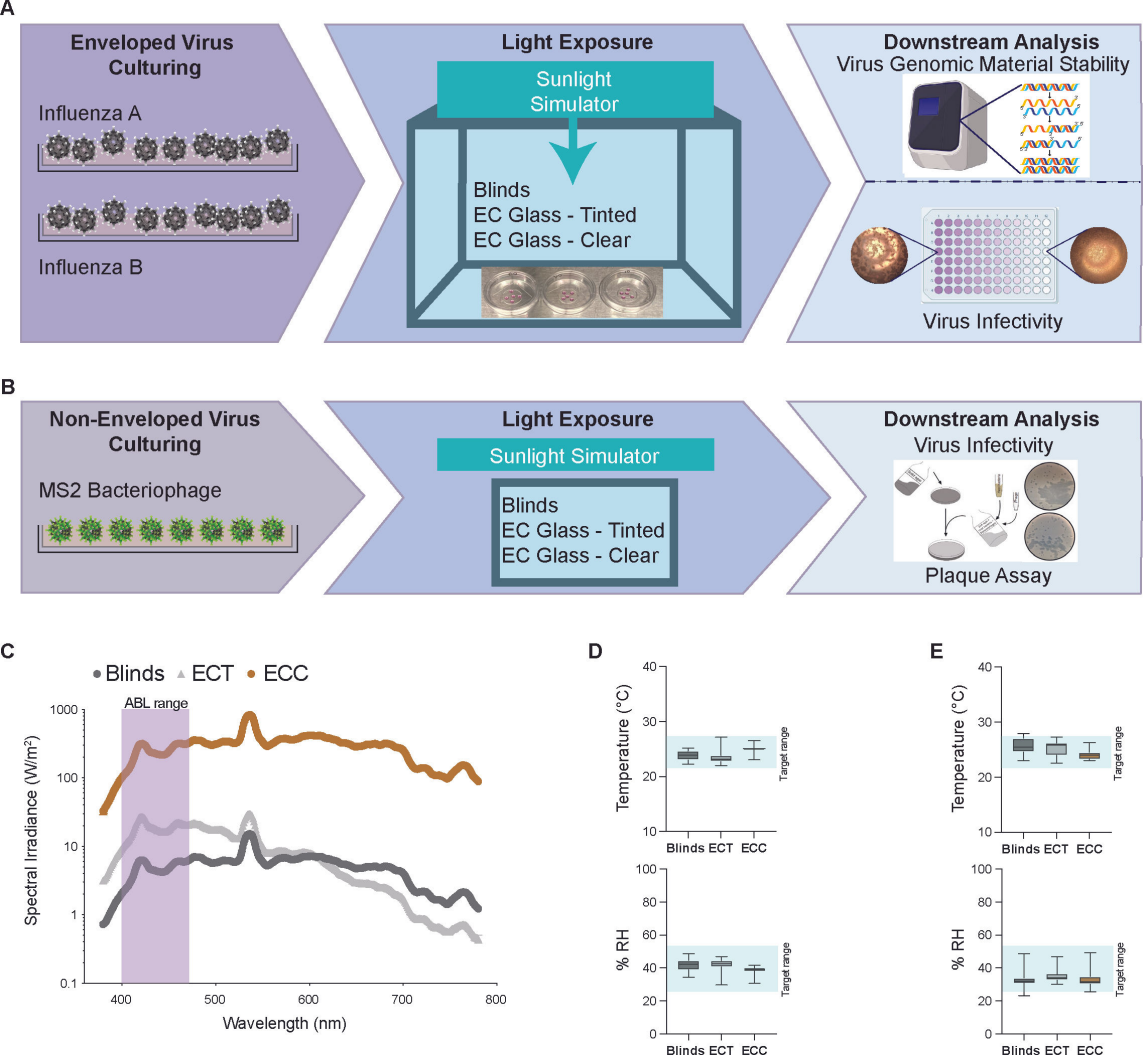
Experimental setup and environmental conditions. (**A and B**) Irradiation workflow showing the design of an environmentally controlled mini-lab chamber with electrochromic (EC) windows and downstream assays for infectivity and genomic stability for the tested enveloped viruses (**A**) and non-enveloped viruses (**B**). (**C**) Line graphs showing mean spectral irradiance measured in the mini-lab chamber at different EC window tints: Blinds (black), electrochromic tinted (ECT) (gray), and electrochromic clear (ECC) (brown). Antimicrobial blue light (ABL), 400–470 nm, spectrum is highlighted with purple. (**D and E**) Box plots showing median ± SEM of temperature (top) and relative humidity (bottom) for influenza A (**D**) and influenza B (**E**). The reference shaded area (light blue) indicates the target range for temperature, 22–28°C, and humidity, 25–55%. Conditions are represented by the following colors: blinds (black), ECT (gray), and ECC (brown).

All experiments were conducted in an environmentally controlled mini-lab cubical chamber setup, where EC windows were mounted on all sides of the chamber around a metal frame (Fig. 1A and B). The tint level of the EC windows was given a control panel on the front of the chamber for manual control. Two side-mounted fans were utilized to simulate indoor room ventilation. A sunlight simulator (Sciencetech) in the form of a xenon arc lamp and a series of optical filters was used to simulate daylight entering the chamber and modulated through the upper EC window panel. Indoor daylight spectrum and intensity were regulated through different EC tinting as well as the placement of blinds using Velcro to simulate the lowering of blinds to block indoor daylight. The different wavelength spectra and intensities between the different tested tint level conditions are shown in Fig. 1C. Temperature and relative humidity were monitored via a HOBO environmental sensor (HOBO MX Analog/Temp/RH/Light [MX1104]). Relative humidity was controlled automatically via a humidifier, and temperature was controlled via an attached air conditioner to maintain a narrow range but allow for realistic fluctuations, temperature, 22–28°C, and humidity, 25–55%, of environmental conditions for testing (Fig. 1D and E).

### Influenza viral stock preparation

Influenza A virus (A/Puerto Rico/8/34 [H1N1], ATCC VR-95) and influenza B virus (B/Florida/78/2015, ATCC VR-1930) stocks were produced and maintained at our laboratory in the University of British Columbia, Kelowna (BC, Canada). Both viruses were separately propagated in cell cultures of Madin-Darby Canine Kidney cells (MDCK, ATCC CCL-34) in growth medium, advanced Dulbecco’s Modified Eagle Medium (DMEM) supplemented with bovine serum albumin (BSA), HEPES-buffered saline solution (2.5%), and 1 µg/mL N-tosyl-L-phenylalanine chloromethyl ketone (TPCK)-trypsin solution (Thermo Scientific), with all other reagents purchased from Gibco Life Sciences. Viruses were harvested by ultracentrifugation with Amicon Ultra-15 ultra centrifugal filters (Sigma-Aldrich). Viral stocks were stored at −80°C prior to the experiments.

### MS2 viral stock preparation

MS2 bacteriophage (ATCC 15597-B1) stocks were produced and maintained in *Escherichia coli* (*E. coli*) host (ATCC 15597) using a double layer agar method. Briefly, Lysogeny Broth (LB) agar was prepared with two layers, 1% (wt/vol) on the bottom and overlaid with a soft top agar layer with 0.6% (wt/vol) agar mixed with 50 µL of *E. coli* host adjusted to an optical density (OD) of 0.5–0.7 at 600 nm. Serial dilutions of MS2 bacteriophage were prepared, spread on the soft agar layer, and incubated at 37°C overnight. Following the incubation, 5 mL of SM buffer (50 mM Tris-HCl at pH 7.5, 100 mM NaCl, 1 mM MgSO_4_, and 0.01% (wt/vol) gelatin) was added on top of the soft agar, and a sterile loop was used to scrape the soft agar into a syringe. The lysate was then filtered using a 0.22 µm filter (Millipore) and stored at −80°C prior to the experiments.

### Irradiance of viruses

Stock suspensions of influenza viruses were taken from −80°C storage and diluted 1:10 in Eagle’s Minimum Essential Medium (EMEM) with 10% fetal bovine serum (FBS). Five 5  µL droplets were placed on glass-bottom Petri dishes to simulate respiratory droplets. For MS2 bacteriophage, stock suspensions were taken from −80°C storage diluted to 10^−10^ PFU/mL in phosphate-buffered saline (PBS). One milliliter was uniformly distributed on Petri dishes. Petri dishes were placed randomly in the environmental chamber and irradiated under ECC, ECT, and Blinds conditions for 8, 12, and 24 h for IVs or 12, 24, and 48 h for MS2 bacteriophage. For all viruses, control samples were those not exposed to any light under the same environmental conditions in the mini-lab chamber. IVs were extracted from the plates by flooding with 250  µL of infection media (EMEM with 10% FBS and 1  µg/mL TPCK-trypsin for enveloped viruses or PBS for non-enveloped viruses) and gently swirling for 2 min, repeated twice to yield 500  µL. MS2 bacteriophage was extracted from the plates by flooding 100 µL of PBS and mixing.

### Genomic stability and infectivity assessment of enveloped viruses

We define viral genomic stability as a metric of genomic material integrity, measured as the relative abundance of viral RNA detected by RT-qPCR, and infectivity reflecting its capacity to remain infectious under specific environmental conditions. For virus genomic stability assessment, virus RNA was extracted using Invitrogen’s PureLink Viral RNA/DNA Mini Kit (Thermo Fisher Scientific), according to the manufacturer’s protocol. For optimal quantitative polymerase chain reaction (qPCR) performance, Ultraplex 1-Step ToughMix 4X (Quantabio), along with probes and primers listed in Table 1, was used (all purchased from Integrated DNA Technologies). QPCR analysis was performed on Bio-Rad CFX Opus system with 40 cycles and a 10 µL reaction volume. The reaction program was set as follows: cDNA synthesis at 95°C for 10 min, preheating at 95°C for 3 min, and amplification at 95°C for 3 s and 55°C for 30 s. Viral RNA reduction following light exposure was quantified using the RT-qPCR data. The percentage reduction compared to the Blinds condition was calculated using the following formula:


$$Percentage\;reduction=\left(\frac{\left(C_{control}-C_{treated}\right)}{C_{control}}\right)\times 100$$

**TABLE 1:** Primers and probes used in influenza A and influenza B quantitative RT-qPCR analysis targeting matrix protein

Oligo function	Primer sequence (5′-3′)	Genomic target
Influenza A		Matrix M gene
Forward primer	CAA GAC CAA TCY TGT CAC CTC TGA C	
Reverse primer	GCA TTY TGG ACA AAV CGT CTA CG	
Probe	/FAM/TGC AGT CCT /ZEN/ CGC TCA CTG GGC ACG/ BHQ1 Q/	
Influenza B		Matrix M gene
Forward primer	TCC TCA AYT CAC TCT TCG AGC G	
Reverse primer	CGG TGC TCT TGA CCA AAT TGG	
Probe	/Cy5/CCA ATT CGA/TAO/ GCA GCT GAA ACT GCG GTG/3IAbRQSp/	

where *C*_control_ is the gene copy number/μL from foiled control samples and *C*_treated_ is the calculated copy number/μL from irradiated samples at the same time point. Gene copy numbers/μL were determined from Cq values using standard curves generated from known RNA concentrations for each virus and are provided in Data S1. Each condition was tested in biological triplicates, and mean percentage reduction values with standard deviations were reported.

Infectivity of viruses was assessed by microtitration using a median tissue culture infectious dose (TCID_50_) assay. In brief, MDCK cells were seeded in 96-well flat bottom plates (5 × 10^4^ cells/100 µL, 100 µL/well) and grown to 80% confluency overnight (37°C, 5% CO_2_). For each experiment, four replicate wells were infected by addition of 100 µL of viral sample per well, then each serially diluted twofold. Following 2 h of incubation, an additional 100 µL of infection media was added to each well. 96-well plates were incubated for 5 days at 37°C and 5% CO_2_ and at 5 days post-infection. Plates were assessed visually for cytopathic effects. TCID_50_/mL was calculated using the method of Spearman and Karber (49). Each condition was tested in biological triplicates, and mean TCID_50_ values with standard deviations are reported in Data S2.

### Infectivity assessment of non-enveloped viruses

Plaque assays were used to assess the effect on MS2 bacteriophage using an *E. coli* host and a double-layer agar method as described in stock preparation. Following irradiance, 100 µL of extracted viruses were plated using the spiral plating method with a sterile cell spreader. The inoculated plates were sealed with Parafilm (Pechiney Plastic Packaging Inc.) and incubated in the dark under static conditions at 37°C overnight. Following incubation, plaque-forming units (PFU) were enumerated manually, and PFU/mL was calculated (data provided in Data S3). The percentage reduction was calculated relative to the PFU of the non-irradiated control group, as described above. Each condition was tested in biological triplicates, and mean percentage reduction values with standard deviations were reported.

### Statistical analysis

All statistical analyses were conducted using R Studio 4.2.2 with 5% risk allowance. Visualizations of data were conducted using GraphPad Prism version 10.0.0. Shapiro-Wilk tests were used to assess the normality of data. Depending on normality, an appropriate test was chosen, either Kruskal-Wallis (for non-normally distributed data) or analysis of variance (ANOVA, for normally distributed data). If the data were determined to be statistically significant, an appropriate post-hoc test was conducted: Kruskal-Wallis followed by Dunn’s test with Holm-Bonferroni adjustment, or ANOVA followed by Tukey Honestly Significant Difference (HSD) with Tukey adjustment.

## RESULTS

Environmental conditions were kept within a narrow range throughout all experiments with an overall average temperature of 24.72 ± 1.37°C and an average relative humidity of 36.65 ± 5.04% (Fig. 1D and E). UVA presence in ECC, ECT, and Blinds was measured to be 83 μW/cm^2^, 5 μW/cm^2^, and 0 μW/cm^2^, respectively. Furthermore, average light intensity (in Lux) in ECC, ECT, and Blinds was measured to be 29,130.79 ± 1,823.44 lx, 488.11 ± 27.33 lx, and 408.73 ± 62.85 lx, respectively. Time-dependent irradiation dosage at 405 nm for ECC, ECT, and Blinds conditions was calculated and is presented in Table S1 as a representative of visible light dosage in the ABL range (Fig. 1C).

### Indoor daylight effect on the stability of genomic material for enveloped influenza viruses

**Fig 2:**
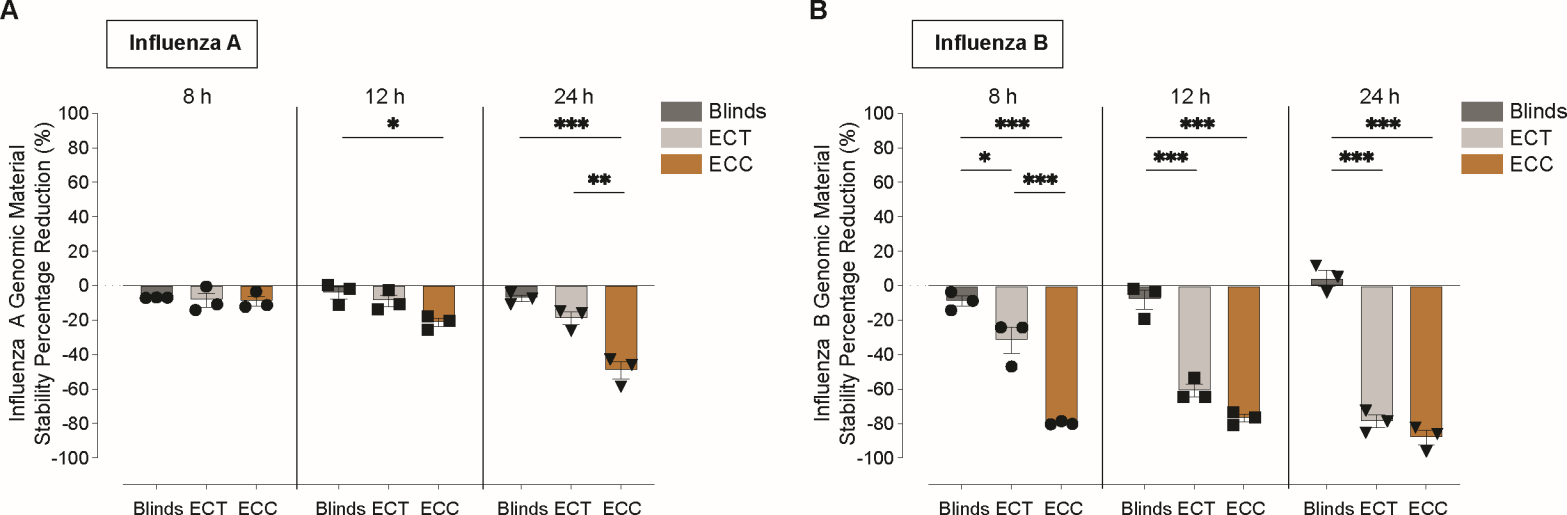
Effect on the stability of genomic material for influenza viruses after indoor daylight exposure at different electrochromic (EC) window conditions using quantitative polymerase chain reaction (qPCR). Influenza A (**A**) and influenza B (**B**) were exposed at Blinds (dark gray), EC tinted (ECT) (gray), and EC clear (ECC) (brown) for 8, 12, and 24 h. Irradiation with percentage reductions compared to non-irradiated controls is shown. Data represent mean ± SEM, with *n* = 3 replicates per condition. Comparisons between groups were performed by one-way analysis of variance (ANOVA) or by Kruskal-Wallis. Statistical significance was considered at *P* < 0.05, where *P < 0.05, **P < 0.01, and ***P < 0.001.

Our results show that IAV exhibited modest reductions in recoverable genomic material under all indoor daylight conditions at 8 h of exposure, with no statistically significant differences between conditions (adj *P*-value > 0.05; Fig. 2A). However, by 12 h, a significant reduction in recovered viral genomic material was observed under the ECC condition compared to the Blinds condition (adj *P*-value = 0.02), indicating enhanced destabilization with higher daylight exposure. Further reductions were noted at 24 h of exposure, showing significant reductions in both ECC and ECT conditions when compared to the Blinds condition (ECC vs Blinds: adj *P*-value < 0.01; ECT vs Blinds: adj *P*-value < 0.01).

In contrast, IBV displayed greater sensitivity to indoor light exposure. Significantly greater reductions of genomic material recovered were detected as early as 8 h under both ECC and ECT conditions compared to Blinds (adj *P*-value < 0.01), and this trend persisted through 12 and 24 h of exposure (ECC vs Blinds: adj *P*-value < 0.01; ECT vs Blinds: adj *P*-value < 0.01; Fig. 2B; Table S2). While ECC vs ECT was significantly different at 8 h of exposure (adj *P*-value = 0.03), they were observed to be non-significant at 12 h (adj *P*-value = 0.07) and 24 h (adj *P*-value = 0.3), as reductions in the ECT condition increased with longer exposure times. Both ECC and ECT led to measurable reductions in recovered IBV genomic material relative to controls, indicating that both light intensity and light spectral composition influence the rate of viral destabilization. These findings highlight that even low-intensity light, such as that in the ECT condition, can significantly reduce IBV genomic stability over time.

### Indoor daylight effect on the infectivity of enveloped influenza viruses

**Fig 3:**
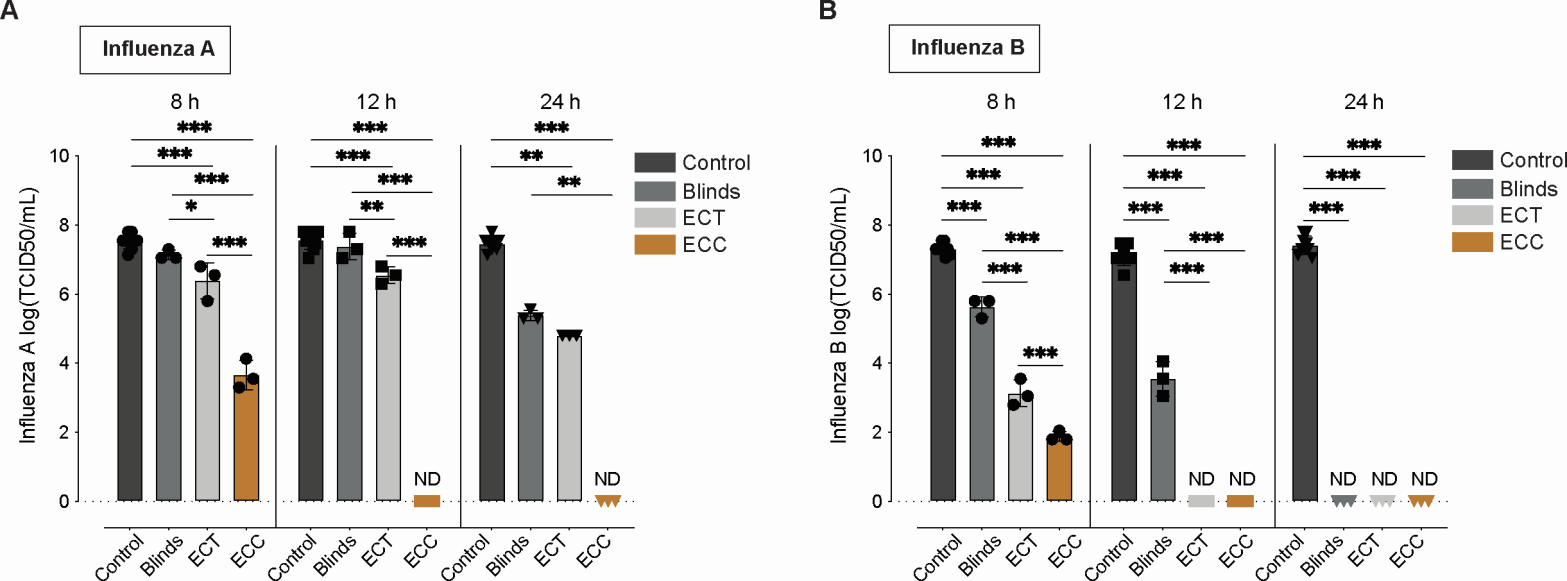
Effect on infectivity of influenza viruses after indoor daylight exposure in different electrochromic (EC) window conditions using a median tissue culture infectious dose (TCID_50_) assay. Log(TCID_50_/mL) of influenza A (**A**) and influenza B (**B**) at control (black), Blinds (dark gray), EC tinted (ECT) (gray), and EC clear (ECC) (brown) are shown at 8, 12, and 24 h of irradiation. Data represent mean ± SEM, with *n* = 3 replicates per condition and *N* = 9 for controls. Not detectable (ND) was used when viral titers dropped below detection limit. Comparisons between groups were performed by one-way analysis of variance (ANOVA) or by Kruskal-Wallis. Statistical significance was considered at *P* < 0.05, where *P < 0.05, **P < 0.01, and ***P < 0.001.

In contrast to our viral genomic stability assessment, IAV infectivity was more sensitive to indoor daylight exposure, particularly under high-intensity light conditions. The strongest reduction was observed under the ECC condition, where IAV infectivity declined by 3.47 log₁₀ TCID₅₀/mL after 8 h (adj *P*-value < 0.01) compared to Blinds and fell below the detection limit of 1.05 log₁₀ TCID₅₀/mL at both 12 and 24 h of exposure (Fig. 3A; Tables S3 and S4).

In the Blinds condition, IAV infectivity remained largely stable at 8 and 12 h with only a modest reduction observed at 24 h (2.07 log₁₀ reduction, adj *P*-value = 0.1, compared to control), indicating that low-light conditions had limited impact on viral infectivity (Fig. 3A).

Under the ECT condition, where light intensity is low, but the spectral composition differs from Blinds, a consistent but minor reduction in infectivity was observed across all time points compared to the control, where no light exposure was applied (Fig. 3A). Specifically, a 1.13 log₁₀ reduction at 8 h (adj *P*-value < 0.01), 1.01 log₁₀ reduction at 12 h (adj *P*-value < 0.01), and 2.66 log₁₀ reduction at 24 h (adj *P*-value = 0.01) were observed (Fig. 3A). When directly compared to the Blinds condition, ECT showed slightly lower viral titers at each time point: a 0.75 log₁₀ reduction at 8 h (adj *P*-value = 0.04), 0.83 log₁₀ reduction at 12 h (adj *P*-value < 0.01), and 0.58 log₁₀ reduction at 24 h (adj *P*-value = 0.46).

In contrast, IBV infectivity showed broader sensitivity to indoor daylight exposure (Fig. 3B). At 8 h, significant reductions in infectivity were observed in both the ECC (3.75 log₁₀ reduction, adj *P*-value < 0.01) and ECT (2.5 log₁₀ reduction, adj *P*-value < 0.01) conditions compared to Blinds. By 12 h, infectious titers were undetectable in both ECC and ECT groups. Notably, even under Blinds, IBV infectivity progressively declined, with titers falling below the detection limit by 24 h.

### Indoor daylight effect on the infectivity of non-enveloped MS2 bacteriophage

**Fig 4:**
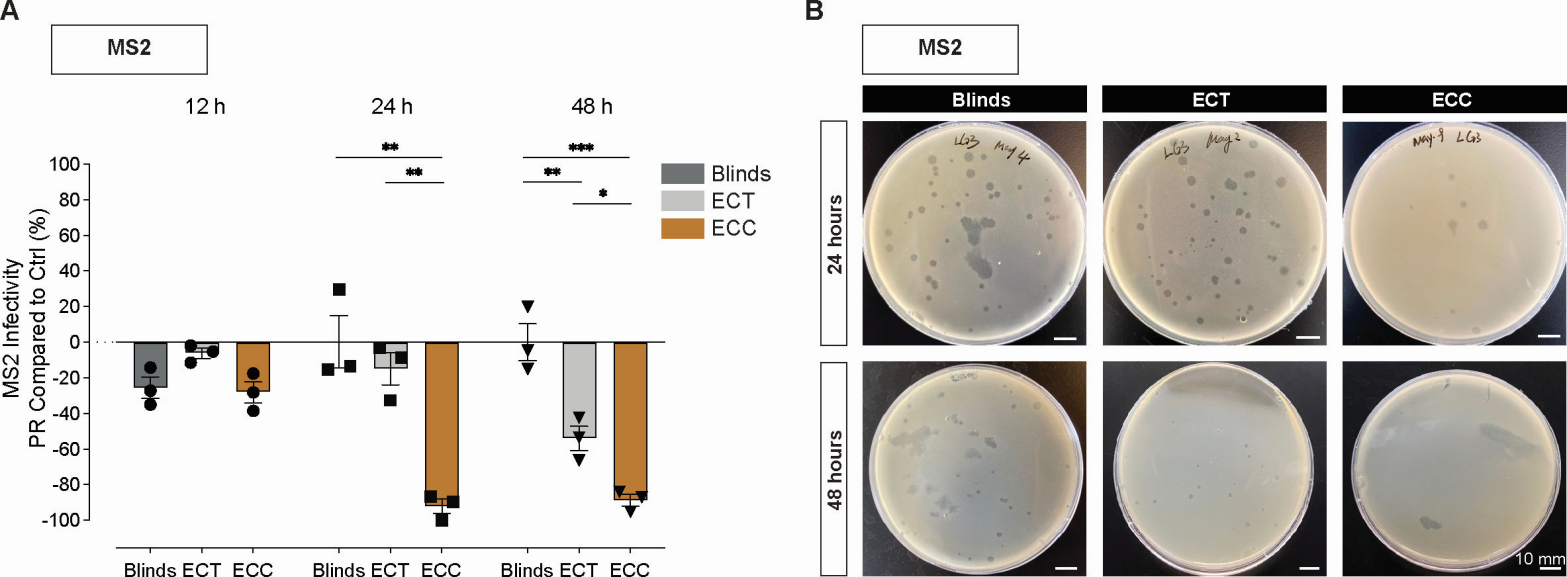
(**A**) Effect on MS2 bacteriophage after indoor daylight exposure at different electrochromic (EC) window conditions using the plaque assay. Plaque-forming units per mL (PFU/mL) were recorded following irradiation at 12, 24, and 48 h of exposure, and percentage reduction (PR) compared to non-irradiated controls was calculated for Blinds (dark gray), EC tinted (ECT) (gray), and EC clear (ECC) (brown). Data represent mean ± SEM, with *n* = 3 replicates per condition. Comparisons between groups were performed by one-way analysis of variance (ANOVA). Statistical significance was considered at *P* < 0.05, where *P < 0.05, **P < 0.01, and ***P < 0.001. (**B**) Representative plate images with plaques on an *E. coli* lawn inoculated with MS2 bacteriophage after 24 and 48 h of exposure at different EC window tints. Scale bars represent 10 mm.

To further assess the antiviral effects of indoor daylight, we evaluated the infectivity of MS2 bacteriophage, a non-enveloped RNA virus, under the same light conditions. Compared to the enveloped influenza viruses, MS2 exhibited greater resistance to light-induced inactivation, with no significant reduction in infectivity observed across any condition after 12 hours of exposure (Fig. 4A; Table S5).

Significant reductions compared to the Blinds condition in MS2 plaque-forming units (PFU) were observed after 24 h of exposure under ECC conditions (adj *P*-value < 0.01), while no change was detected in either the Blinds or ECT groups at that time point. By 48 h, PFU counts were significantly reduced under both ECC (adj *P*-value < 0.01) and ECT (adj *P*-value < 0.01) compared to Blinds, although inactivation was more pronounced under ECC. The Blinds condition did not produce any significant loss of MS2 infectivity, even at the longest exposure duration compared to controls.

These results suggest that MS2 is more resistant to indoor daylight exposure than enveloped viruses, particularly during early exposure. However, extended exposure to high-intensity daylight (ECC) led to substantial inactivation, and even low-intensity light with altered spectral composition (ECT) resulted in reduced infectivity over longer periods. This highlights the importance of both duration and light characteristics in achieving antiviral effects against more resilient, non-enveloped viruses.

## DISCUSSION

Our results indicate that indoor daylight modulation can exert antiviral effects across a range of virus types, but its effectiveness depends critically on the interplay between light spectrum, intensity, and duration of exposure. IBV was consistently the most sensitive virus across all light conditions, while in contrast, IAV demonstrated greater resilience but was still susceptible to light-induced inactivation under specific conditions. Specifically, for IAV, infectivity under high-intensity light (ECC) declined by 1.13 log₁₀ reduction after 8 h and by 2.66 log₁₀ reduction at 24 h (Fig. 3A), while genomic stability remained relatively preserved until a modest reduction compared to linds emerged at 12 h (Fig. 2A). While indoor daylight spectrum changes to blue-enriched light (ECT) had a modest effect on IAV, its effect on IBV was more pronounced, with significant decreases observed in both infectivity and genomic material recovered as early as 8 h of exposure. Furthermore, for IBV, low-intensity blue-enriched light (ECT) did not have a significantly different effect from high-intensity (ECC) light on both infectivity and genomic stability after 12 h, indicating that spectrum was likely the dominant factor for photoinactivation in IBV. Moreover, when these observations are put into the perspective of longitudinal exposure, they suggest that early-stage photodamage to envelope glycoproteins from low-intensity light (50) may precede genomic degradation and that IAV requires higher intensity exposure to achieve meaningful inactivation. Furthermore, consistent with previous studies on blue-light photoinactivation (36, 51), the non-enveloped virus MS2 bacteriophage was the most resistant virus tested when extended into the indoor setting with daylight modulation. These results underscore the role of viral structure in determining susceptibility to daylight-mediated inactivation.

Mechanistically, photoinactivation is believed to involve the generation of reactive oxygen species (ROS) by endogenous photosensitizers incorporated into viral membranes during assembly (51, 52). These ROS can damage surface proteins and lipid envelopes before affecting nucleic acids, consistent with our observation that infectivity typically declined before genome degradation (53). The absence of a lipid envelope in MS2 likely confers higher resistance to photoinactivation, as previously shown for non-enveloped viruses, which lack the oxidative targets typically found in viral membranes (36, 51). Our findings extend this understanding by revealing that even within this group, strain-level differences significantly affect photoreactivity. IBV and IAV differ in membrane lipid composition, membrane proteins, genome length, and possibly capsid architecture, factors that influence how each respond to photonic stress (54–56). These structural distinctions likely explain why IBV is more readily inactivated than IAV, even under comparable light conditions. While the data are sparse, similar findings regarding different reactions of IBV and IAV to light have been reported previously with a different IBV subtype (57), thus possibly suggesting that the current study’s findings may be generalizable to a certain degree at the strain level. However, it is important to note that UV-light inactivation studies have revealed that even within strains, there may be differential effects (41); however, such findings have not yet been fully extended to visible light and ABL. While we only examined viruses different at the strain-level, such differences between and within strains are often overlooked in light-based disinfection strategies, yet they are critical to designing interventions that reflect real-world viral diversity.

While our data clearly show that the antiviral effects of indoor daylight vary by virus type, they also point to a practical opportunity: preserving blue-enriched light indoors may help reduce the environmental persistence of influenza B virus (IBV) on surfaces. IBV was markedly more susceptible than influenza A virus (IAV) under both low- and high-intensity conditions, suggesting that light-based interventions could be especially beneficial during IBV-dominant phases of the flu season.

These findings gain additional relevance when considered alongside recent surveillance trends. Although influenza A viruses typically lead early-season circulation in the United States, IBV has shown a consistent pattern of increased activity later in the season. For instance, during the 2023–2024 flu season, the B/Victoria lineage became a leading subtype in early spring, following a decline in A(H1N1) activity (58, 59). This shift in timing is particularly significant for high-occupancy settings, such as schools and workplaces, where daylight access can be limited and late-season outbreaks can be especially disruptive.

Given that IBV disproportionately affects children and young adults, and that its seasonal dynamics do not always mirror those of influenza A (6, 8, 58, 59), there is growing motivation to consider environmental factors that may influence its transmission. Our findings suggest that indoor lighting strategies preserving key parts of the visible spectrum, particularly the blue-light range, could serve as a passive, yet practical, addition to existing infection control efforts. Implementing such strategies in architectural design or public health policy may help mitigate transmission risk during periods of heightened IBV circulation, particularly in the latter part of the flu season.

These strategies, however, must be contextualized within the broader geographic and seasonal landscape that shapes daylight availability and viral inactivation potential. Regions with high sun angles and extended daylight hours during summer may naturally benefit from increased exposure to antimicrobial wavelengths. In contrast, high-latitude areas and urban environments with limited solar access may require more intentional light modulation, especially during winter months when indoor transmission tends to rise (60–63). Notably, lower solar angles in winter can also enhance the penetration of light indoors, coinciding with flu season peaks and offering an opportunity to use daylight more strategically.

Finally, although this study focused on surface-deposited viruses in a controlled setting that allowed us to control the testing environment and focus on specific light exposures, this does not fully reflect the multiple ways IVs are often transmitted, which also include airborne transmission in aerosol form. Biosafety and technical constraints limited our ability to perform photoinactivation studies in aerosolized systems. Additionally, we used glass as the primary surface material to support consistent transmission of daylight and minimize variability introduced by surface properties. However, we acknowledge that other materials, such as plastic, wood, and stainless steel, are far more representative of surfaces encountered in indoor settings. These materials may interact differently with light and could influence the effectiveness of photoinactivations. Future studies should address these gaps by exploring the efficacy of indoor daylight and ABL in inactivating aerosolized viruses under realistic environmental conditions and diverse material types. Nonetheless, these findings support the potential role of indoor daylight, when thoughtfully preserved and spectrally enriched, as part of a layered approach to reduce viral persistence in built environments.

## Data Availability

The data generated in this study are available in the presented figures, tables, and supplemental material. Material requests should be addressed to Dr. Sepideh Pakpour, sepideh.pakpour@ubc.ca.

## SUPPLEMENTAL MATERIAL

Data S1 aem.00973-25-s0001.xlsx Raw data and analysis of relative abundance of influenza virus RNA detected by RT-qPCR.

Data S2 aem.00973-25-s0002.xlsx Raw data and analysis of log(TCID50/mL) of influenza viruses detected by TCID50 assay.

Data S3 aem.00973-25-s0003.xlsx Raw data and analysis of PFU/mL of MS2 detected by plaque assay.

Supplemental tables aem.00973-25-s0004.docx Tables S1 to S5.

ASM does not own the copyrights to Supplemental Material that may be linked to, or accessed through, an article. The authors have granted ASM a non-exclusive, world-wide license to publish the Supplemental Material files. Please contact the corresponding author directly for reuse.
