# Supplementary material for: Photoinactivation of influenza viruses by modulated indoor daylight spectrum and intensity
Source: Appl Environ Microbiol. 2025 Oct 2;91(10):e00973-25. doi: 10.1128/aem.00973-25 (PMC12542662; doi:10.1128/aem.00973-25)
Supplement: Supplemental tables — Tables S1 to S5. [file aem.00973-25-s0004.docx]

**Supplementary Materials**

**Supplementary Data 1.** Raw data and analysis of relative abundance of Influenza viruses RNA detected by RT-qPCR.

**Supplementary Data 2.** Raw data and analysis of log(TCID50/mL) of Influenza Viruses detected by TCID50 Assay.

**Supplementary Data 3.** Raw data and analysis of PFU/mL of MS2 detected by Plaque Assay.

**Supplementary Table 1**. Time-dependent irradiation dosages at different electrochromic window conditions, Electrochromic Clear (ECC), Electrochromic Tinted (ECT), and Blinds, recorded at 405 nm.

| EC Condition | Irradiation Dosage (W/cm^2^) | 8 Hours Dosage (J/cm^2^) | 12 Hours Dosage (J/cm^2^) | 24 Hours Dosage (J/cm^2^) | 48 Hours Dosage (J/cm^2^) |
| --- | --- | --- | --- | --- | --- |
| ECC | 0.0397 | 1143.36 | 1715.04 | 3430.08 | 6860.16 |
| ECT | 0.000180 | 5.18 | 7.78 | 15.55 | 31.10 |
| Blinds | 0.0000694 | 2.00 | 3.00 | 6.00 | 12.00 |

**Supplementary Table 2**. Statistical significance of the genomic stability of Influenza viruses after indoor daylight exposure to different electrochromic window conditions, Electrochromic Clear (ECC) Electrochromic Tinted (ECT), and Blinds. Post-hoc comparisons were only conducted if the initial statistical test yielded a statistically significant result (p<0.05).

| Species | Time | Test | Post-Hoc | Comparison | *p-value* |
| --- | --- | --- | --- | --- | --- |
| Influenza A | 8 hours | Kurskal | N/A | N/A | 0.73 |
| Influenza A | 12 hours | ANOVA | TukeyHSD | Blinds - ECC | 0.02 |
| Influenza A | 12 hours | ANOVA | TukeyHSD | Blinds - ECT | 0.57 |
| Influenza A | 12 hours | ANOVA | TukeyHSD | ECT - ECC | 0.06 |
| Influenza A | 24 hours | ANOVA | TukeyHSD | Blinds - ECC | <0.001 |
| Influenza A | 24 hours | ANOVA | TukeyHSD | Blinds - ECT | 0.15 |
| Influenza A | 24 hours | ANOVA | TukeyHSD | ECT - ECC | 0.003 |
| Influenza B | 8 hours | ANOVA | TukeyHSD | Blinds - ECC | <0.001 |
| Influenza B | 8 hours | ANOVA | TukeyHSD | Blinds - ECT | 0.03 |
| Influenza B | 8 hours | ANOVA | TukeyHSD | ECT - ECC | <0.001 |
| Influenza B | 12 hours | ANOVA | TukeyHSD | Blinds - ECC | <0.001 |
| Influenza B | 12 hours | ANOVA | TukeyHSD | Blinds - ECT | <0.001 |
| Influenza B | 12 hours | ANOVA | TukeyHSD | ECT - ECC | 0.07 |
| Influenza B | 24 hours | ANOVA | TukeyHSD | Blinds - ECC | <0.001 |
| Influenza B | 24 hours | ANOVA | TukeyHSD | Blinds - ECT | <0.001 |
| Influenza B | 24 hours | ANOVA | TukeyHSD | ECT - ECC | 0.32 |

**Supplementary Table 3**. Average log-reductions of Influenza A and Influenza B titers after indoor daylight exposure at different electrochromic window conditions, Electrochromic Clear (ECC) and Electrochromic Tinted (ECT) compared to Blinds condition. Samples that dropped below the detection limit are not shown.

| Species | Time | Comparison | Log-reduction |
| --- | --- | --- | --- |
| Influenza A | 8 hours | Blinds - ECC | 3.47 |
| Influenza A | 8 hours | Blinds - ECT | 0.75 |
| Influenza A | 12 hours | Blinds - ECC | 7.38 |
| Influenza A | 12 hours | Blinds - ECT | 0.83 |
| Influenza A | 24 hours | Blinds - ECC | 5.38 |
| Influenza A | 24 hours | Blinds - ECT | 0.58 |
| Influenza B | 8 hours | Blinds - ECC | 3.75 |
| Influenza B | 8 hours | Blinds - ECC | 2.50 |
| Influenza B | 12 hours | Blinds - ECC | 3.55 |
| Influenza B | 12 hours | Blinds - ECC | 3.55 |

**Supplementary Table 4**. Statistical significance of infectivity of Influenza viruses after indoor daylight exposure at different electrochromic window conditions, Electrochromic Clear (ECC) Electrochromic Tinted (ECT), and Blinds. Post-hoc comparisons were only conducted if the initial statistical test yielded a statistically significant result (p<0.05).

| Species | Time | Test | Post-Hoc | Comparison | *p-value* |
| --- | --- | --- | --- | --- | --- |
| Influenza A | 8 hours | ANOVA | TukeyHSD | Blinds - ECC | <0.001 |
| Influenza A | 8 hours | ANOVA | TukeyHSD | Blinds - ECT | 0.04 |
| Influenza A | 8 hours | ANOVA | TukeyHSD | Blinds - Control | 0.27 |
| Influenza A | 8 hours | ANOVA | TukeyHSD | ECC - Control | <0.001 |
| Influenza A | 8 hours | ANOVA | TukeyHSD | ECT - Control | <0.001 |
| Influenza A | 8 hours | ANOVA | TukeyHSD | ECT - ECC | <0.001 |
| Influenza A | 12 hours | ANOVA | TukeyHSD | Blinds - ECC | <0.001 |
| Influenza A | 12 hours | ANOVA | TukeyHSD | Blinds - ECT | 0.008 |
| Influenza A | 12 hours | ANOVA | TukeyHSD | Blinds - Control | 0.72 |
| Influenza A | 12 hours | ANOVA | TukeyHSD | ECC - Control | <0.001 |
| Influenza A | 12 hours | ANOVA | TukeyHSD | ECT - Control | <0.001 |
| Influenza A | 12 hours | ANOVA | TukeyHSD | ECT - ECC | <0.001 |
| Influenza A | 24 hours | Kruskal | Dunn’s Test | Blinds - ECC | 0.005 |
| Influenza A | 24 hours | Kruskal | Dunn’s Test | Blinds - ECT | 0.46 |
| Influenza A | 24 hours | Kruskal | Dunn’s Test | Blinds - Control | 0.10 |
| Influenza A | 24 hours | Kruskal | Dunn’s Test | ECC - Control | <0.001 |
| Influenza A | 24 hours | Kruskal | Dunn’s Test | ECT - Control | 0.01 |
| Influenza A | 24 hours | Kruskal | Dunn’s Test | ECT - ECC | 0.46 |
| Influenza B | 8 hours | ANOVA | TukeyHSD | Blinds - ECC | <0.001 |
| Influenza B | 8 hours | ANOVA | TukeyHSD | Blinds - ECT | <0.001 |
| Influenza B | 8 hours | ANOVA | TukeyHSD | Blinds - Control | <0.001 |
| Influenza B | 8 hours | ANOVA | TukeyHSD | ECC - Control | <0.001 |
| Influenza B | 8 hours | ANOVA | TukeyHSD | ECT - Control | <0.001 |
| Influenza B | 8 hours | ANOVA | TukeyHSD | ECT - ECC | <0.001 |
| Influenza B | 12 hours | ANOVA | TukeyHSD | Blinds - ECC | <0.001 |
| Influenza B | 12 hours | ANOVA | TukeyHSD | Blinds - ECT | <0.001 |
| Influenza B | 12 hours | ANOVA | TukeyHSD | Blinds - Control | <0.001 |
| Influenza B | 12 hours | ANOVA | TukeyHSD | ECC - Control | <0.001 |
| Influenza B | 12 hours | ANOVA | TukeyHSD | ECT - Control | <0.001 |
| Influenza B | 12 hours | ANOVA | TukeyHSD | ECT - ECC | 1 |
| Influenza B | 24 hours | ANOVA | TukeyHSD | Blinds - ECC | 1 |
| Influenza B | 24 hours | ANOVA | TukeyHSD | Blinds - ECT | 1 |
| Influenza B | 24 hours | ANOVA | TukeyHSD | Blinds - Control | <0.001 |
| Influenza B | 24 hours | ANOVA | TukeyHSD | ECC - Control | <0.001 |
| Influenza B | 24 hours | ANOVA | TukeyHSD | ECT - Control | <0.001 |
| Influenza B | 24 hours | ANOVA | TukeyHSD | ECT - ECC | 1 |

**Supplementary Table 5**. Statistical significance of effect on MS2 bacteriophage after indoor daylight exposure at different electrochromic window conditions, Electrochromic Clear (ECC) Electrochromic Tinted (ECT), and Blinds. Post-hoc comparisons were only conducted if the initial statistical test yielded a statistically significant result (p<0.05).

| Species | Time | Test | Post-Hoc | Comparison | *p-value* |
| --- | --- | --- | --- | --- | --- |
| MS2 | 12 hours | ANOVA | TukeyHSD | Blinds - ECC | 0.94 |
| MS2 | 12 hours | ANOVA | TukeyHSD | Blinds - ECT | 0.09 |
| MS2 | 12 hours | ANOVA | TukeyHSD | ECT - ECC | 0.06 |
| MS2 | 24 hours | ANOVA | TukeyHSD | Blinds - ECC | 0.002 |
| MS2 | 24 hours | ANOVA | TukeyHSD | Blinds - ECT | 0.58 |
| MS2 | 24 hours | ANOVA | TukeyHSD | ECT - ECC | 0.004 |
| MS2 | 48 hours | ANOVA | TukeyHSD | Blinds - ECC | <0.001 |
| MS2 | 48 hours | ANOVA | TukeyHSD | Blinds - ECT | 0.005 |
| MS2 | 48 hours | ANOVA | TukeyHSD | ECT - ECC | 0.04 |
